# Influence of monsoonal winds on chlorophyll-*α* distribution in the Beibu Gulf

**DOI:** 10.1371/journal.pone.0191051

**Published:** 2018-01-12

**Authors:** Chunyan Shen, Yunrong Yan, Hui Zhao, Jiayi Pan, Adam T. Devlin

**Affiliations:** 1 College of Fisheries, Guangdong Ocean University, Zhanjiang, China; 2 Center of South China Sea Fisheries Resources Monitoring and Assessment, Guangdong Ocean University, Zhanjiang, China; 3 Faculty of Chemistry and Environmental Science, Guangdong Ocean University, Zhanjiang, China; 4 Institute of Space and Earth Information Science, The Chinese University of Hong Kong, Hong Kong, China; 5 Shenzhen Research Institute, The Chinese University of Hong Kong, Shenzhen, China; 6 College of Marine Science, Nanjing University of Information Science and Technology, Nanjing, Jiangsu, China; Fisheries and Oceans Canada, CANADA

## Abstract

The influence of seasonal, monsoonal winds on the temporal and spatial variability of chlorophyll-*a* (chl-*a*) in the Beibu Gulf is studied based on long-term satellite data of sea surface winds, chl-*a* concentration and sea surface temperature (SST) and in-situ observations for the years from 2002 to 2014. The analysis results indicated that under northeasterly monsoonal winds, chl-*a* concentrations were substantially elevated in most area of the Beibu Gulf, with a high chl-*a* concentration (>2 mg m^-3^) patch extending southwestward from the coastal water of the northeastern Gulf, consistent with the winter wind pattern. Meanwhile, the spatial distribution of high chl-*a* concentration is correlated with low SST in the northeastern Gulf. In the southern Gulf, there was generally low chl-*a*, except in the coastal waters southwest of Hainan Island. Here, the upwelling cold water prevails outside the mouth of the Beibu Gulf, driven by the southwesterly monsoonal winds and the runoff from the Changhua River, as implied by low observed SST. Correlation analysis indicated the chl-*a* concentration was strongly modulated by wind speed (r = 0.63, p<0.001), particularly in the middle of the northern Gulf and southern Hainan Island (r>0.7, p<0.001). Integrated analysis also showed that stratification is weak and mixing is strong in winter as affected by the high wind speed, which suggests that the wind-induced mixing is a dominant mechanism for entrainment of nutrients and the spatial distribution of chl-*a* in winter.

## Introduction

Annual atmospheric and upper ocean variability in the SCS region are primarily controlled by the East Asian monsoon, with strong northeasterly monsoonal winds prevailing from September to April, and weak southwesterly monsoonal winds present from July to August [1–3]. Previous studies have confirmed that the East Asian monsoon plays an important role in regulating the spatial distribution of chl-*a* in the SCS [1, 4–6]. Most of the SCS is oligotrophic, with upwelling and vertical mixing induced by the southwest monsoon as primary dynamic mechanisms that deliver sufficient nutrients for phytoplankton growth [7–9], together with the upwelling caused by the northeast monsoon in parts of the SCS northwest of the Luzon Strait [1, 10–11].

**Fig 1:**
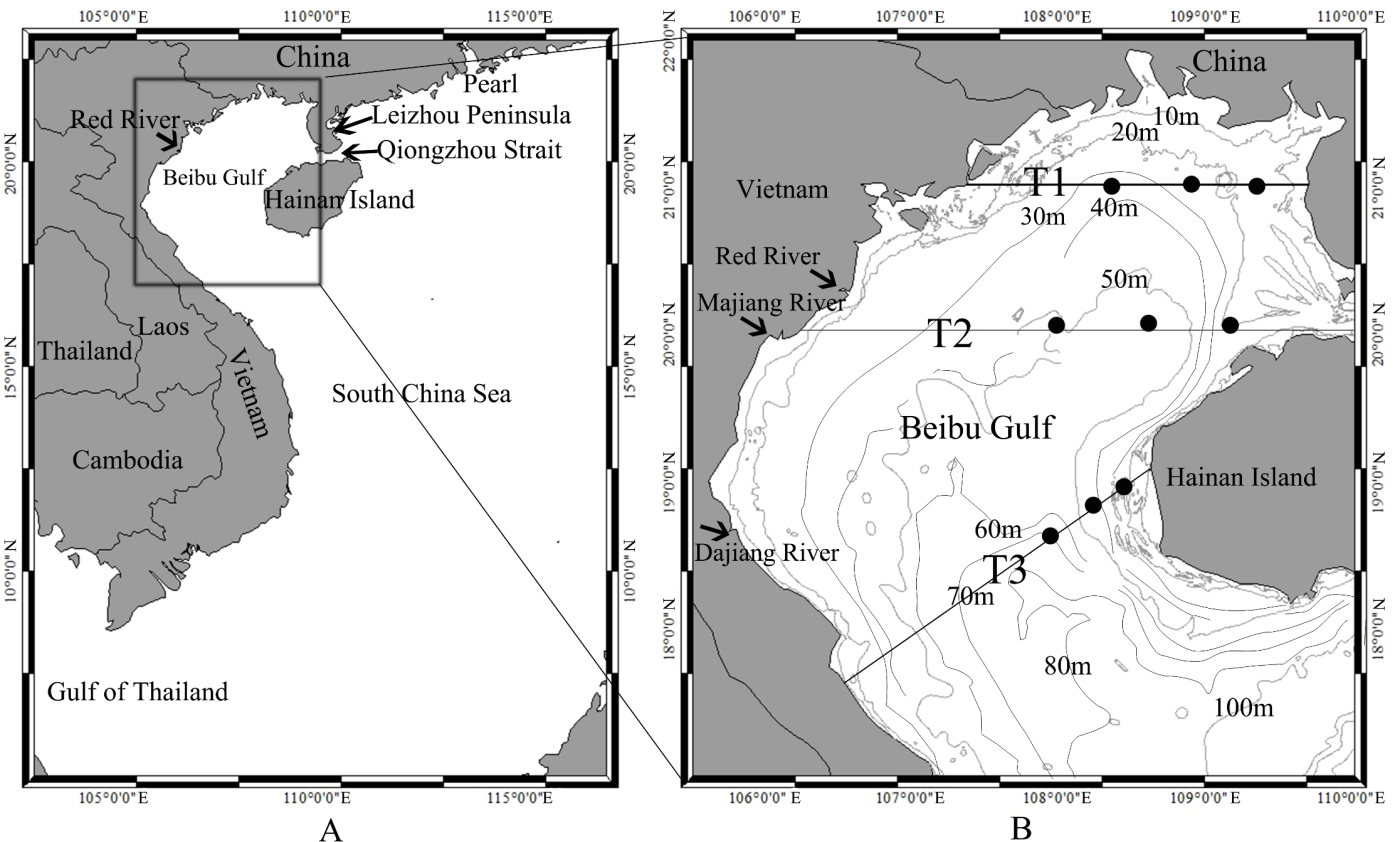
**A: Map of the region of interest, with detailed study region shown by the expanded box. B: The bathymetric chart of the in situ and satellite data sampling area (The base maps of A and B were produced by ARCGIS 10).** The in-situ sampling stations are denoted by dots, and the three transects T1, T2 and T3 are indicated by lines. The path of T1 covers areas with water depth less than 40 m, T2 with water depth less than 60 m, and T3 with water depth greater than 70 m at the mouth of the Beibu Gulf.

The Beibu Gulf, also known as the Gulf of Tonkin, is located in the northwest South China Sea (SCS), centered on the region of 17°-22°N, 105°40′-110°E (Fig 1), and is the home of the fourth largest fishery field in China [12]. The Beibu Gulf is a semi-enclosed water body with complex hydrodynamics, connecting to the main SCS through the south entrance of the Gulf and the Qiongzhou Strait. To the west of the Gulf is Vietnam, and to the east are the Leizhou Peininsula and Hainan Island of China. The isobaths of the Beibu Gulf are generally parallel to the coastline, with a water depth that ranges from 0 to 100 m. There are about 300 rivers flowing into the Beibu Gulf, with a cumulative annual runoff of 1500–2000×10^8^ m^3^ [13]. The major rivers include the Changhua River and the Zhubi River of Hainan, the Nanliu River and the Qinjiang River of Guangxi, and the Red River, the Majiang River and Dajiang River of Vietnam. The Red River alone accounts for 75% of the total runoff [13]. The river water discharges into the Gulf with abundant fresh water and nutrients, which may significantly influence water temperature, salinity, circulation, and phytoplankton growth, especially in coastal regions [14–15].

The weather and marine ecosystem of the Beibu Gulf are greatly affected by the East Asian monsoon, similar to what is seen in the SCS and the Arabian Sea. Biological and ecological studies in the Beibu Gulf were first undertaken in the 1960s, though most of the studies were limited in spatial sampling to coastal regions rather than the whole Gulf. Since the dawn of the satellite technology era in the last century, satellite remote sensing has become an ideal method for large spatial-scale ocean studies, due to the extensive spatial coverage provided by space-borne observation platforms. Seasonal variations of chl-*a* in the Beibu Gulf observed with both in-situ and satellite data have revealed that high pigment concentrations are apparent in the northeast Beibu Gulf around Hainan Island [12,8,16,17]. Satellite images indicate that phytoplankton blooms appear in the central Gulf during the northeasterly winds of the winter monsoon [17], and the seasonal variability of chl-*a* concentration and SST may be associated with seasonally reversing monsoonal winds [8]. Moreover, it was found that northeasterly winter monsoonal winds may induce strong upwelling in the eastern Beibu Gulf along the west side of Hainan Island, and in summer, coastal upwelling due to the southwestern monsoon may occur in the western Gulf [8,17].

The influence of monsoonal winds on phytoplankton variation is significant in the Beibu Gulf. However, the detailed connection between the monsoonal winds and the chl-*a* concentration has been poorly understood as the complex hydrodynamics in the Beibu Gulf as well as the coupling between wind, upwelling, coastal currents and vertical mixing in this area is still unclear. This study utilizes remote sensing and statistical analysis methods to investigate temporal and spatial variability of chl-*a* in the Beibu Gulf, and to suggest some potential drivers leading to the observed variability.

## Materials and methods

### Study area and sampling stations

The large-scale and detailed study areas are shown in Fig 1A and 1B. The lines labelled T1, T2, and T3 represent cruise transects for sampling temperature profiles in different areas of the Beibu Gulf. T1 passed through coastal shallow water regions in the north of the Gulf that are affected by river runoffs and strong tidal currents, T2, in the middle of the Gulf, is less influenced by river runoffs, and T3 in the south of the Gulf is affected by both river runoffs from Hainan Island and denser water from the SCS.

### Satellite and in-situ data

#### Sea surface wind vectors

Ocean surface winds is obtained from the daily WindSat Polarimetric Radiometer data provided by the Remote Sensing Systems in Santa Rosa, California (http://www.remss.com/missions/windsat/), covering the time period of February 2003 to August 2014, with a spatial resolution of 0.25° by 0.25°. However, there are two gaps for the wind data from February 2005 to May 2005 and from June 2007 to July 2007.

#### Chl-*a* and SST

Satellite chl-*a* and SST data are derived from the Moderate Resolution Imaging Spectrometer (MODIS) Aqua mission (https://oceandata.sci.gsfc.nasa.gov/). The monthly L3 product of chl-*a* and SST, with a spatial resolution of 4 km, is obtained for the period from July 2002 to December 2014. In situ chl-*a* data were collected at the surface (1 m) in four cruise surveys conducted in November 2013, February 2014, May 2014, and August 2014 in the study area shown in Fig 1B. Each cruise covered all three transects. A total of nine sampling stations were located along each of the three cruise transects. The samples were filtered through 0.7 um Whatman GF/F glass fibre filters (25 mm) immediately after sampling, and then the filters were stored in aluminium foil and kept at -20°C. Filters with chl-a were extracted with 90% acetone and sonicated for 10 minutes, and then extracted at 40°C in the dark for 24 hours. The fluorescence method was used to measure the chl-a concentration with a Turner Designs model 10-AU fluorometer within 15 days from the sampling date [17, 18, 19]. These in-situ data are used to validate the MODIS-derived chl-*a* concentrations. For the comparison between the satellite and in-situ data in the Beibu Gulf, we selected MODIS L2 data (1 km) taken from the same locations as in-situ data (9 sampling stations in Fig 1B) on the same day. Average values of MODIS L2 chl-a concentration were calculated on 20×20 pixels (that is 20×20 km^2^) for each station, because single scene images do not have good data coverage. A total of 36 pairs of MODIS L2 chl-*a* concentration and the concurrent in-situ data are selected.

#### Mixed layer depth (MLD)

When ocean mixing reaches the sea bottom (i.e. MLD is equal to the water depth.), satellite Chl-*a* products are probably contaminated due to re-suspended sediments from the sea floor, which influence quality of Chl-*a* data retrieval. In order to evaluate the possible influence of re-suspended material (SM) from the sea bottom on chl-*a* concentration observed by satellite sensors, the World Ocean Atlas 2013 dataset (WOA 13) (https://www.nodc.noaa.gov/OC5/WOA94/mix.html) is employed to generate monthly climatological MLD. From salinity and temperature, variable potential density is first calculated to estimate the monthly climatologies MLD using a method similar to that used in [20, 21] in which one starts at a depth of 1 m, and searches down the water column until the potential density has increased by a value over 0.1 kg m^-3^. The MLD based on variable density criterion is designed to account for the large variability of the coefficient of thermal expansion that characterizes seawater.

#### Vertical temperature profiles derived from CTD and CATSAT

**Fig 2:**
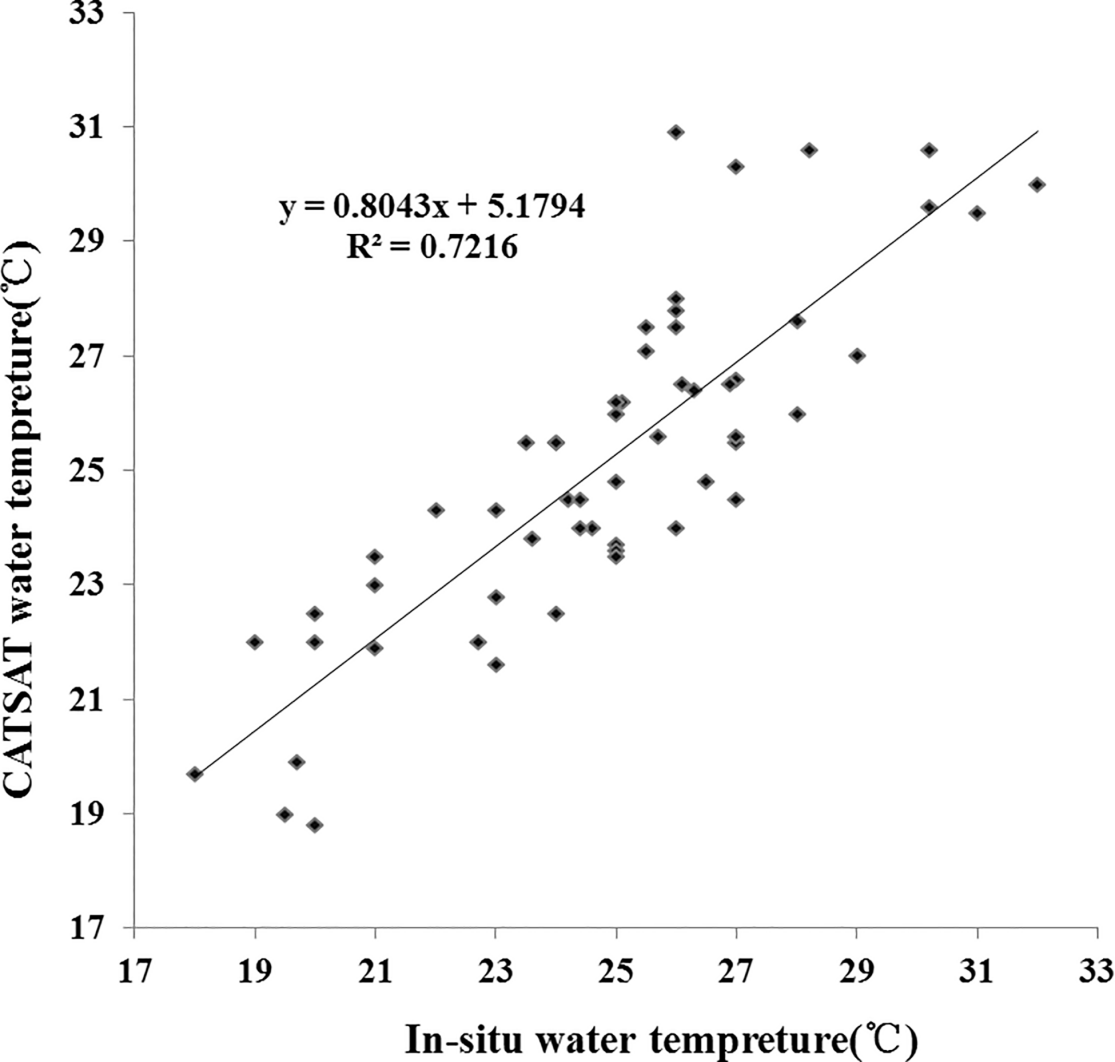
Comparison between CATSAT-derived and in-situ measurements of water temperature.

Different water-level temperature data can provide details about the evolution of oceanic thermal fronts and upwelling events, identifying their affected regions. In this study, water temperature profiles were measured using a Sea-Bird Conductivity-Temperature-Depth (CTD) instrument at the nine sampling stations (Fig 1B). In addition, merged temperature data of the Beibu Gulf are used, which are available in the CATSAT system from Center of South China Sea Fisheries Resources Monitoring and Assessment, Guangdong Ocean University. CATSAT is a key decision tool for professional fishermen, providing near real-time and accurate oceanographic and marine meteorological information. In the CATSAT system, over 40 scientifically-validated ocean data products are included, which merge remote sensing images, buoy and cruise data, and fishing production data. The main CATSAT dataset includes temperature, ocean currents, marine weather, plankton concentration, and salinity, which combines buoy, cruise data and model outputs, at depths of 10, 20, 30, 50, 75, and 100 m, with a spatial resolution of 0.25° by 0.25°. In this study, CATSAT data were used to analyze monthly sub-surface temperatures during the period corresponding with the cruise surveys in the Beibu Gulf. The data were used to generate monthly data from the daily temperature images, after removing spurious pixels associated with data flags (e.g., cloud/stray light, large solar/sensor angle, high aerosol optical thickness). CATSAT daily vertical temperature data are also selected at the CTD sampling locations along the three transects with water depths of 0-80m, within one day of the in-situ observations. In total, 56 matching data pairs were obtained. The two data sets show good agreement (r = 0.85; Fig 2), supporting the use the CATSAT data in this study.

## Results

### Seasonal variation of monthly wind vectors

**Fig 3:**
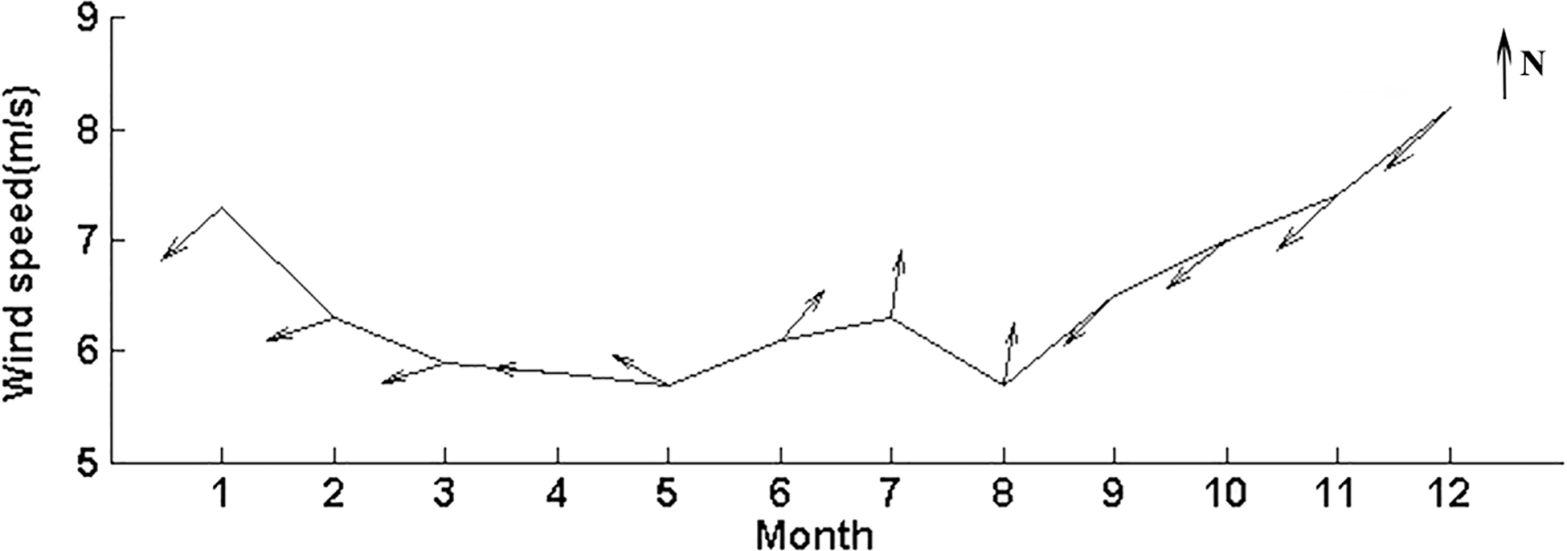
Climatological monthly averaged wind velocity in the Beibu Gulf during July 2002—December 2014. The arrow indicates wind direction, with the northward being upwards.

**Fig 4:**
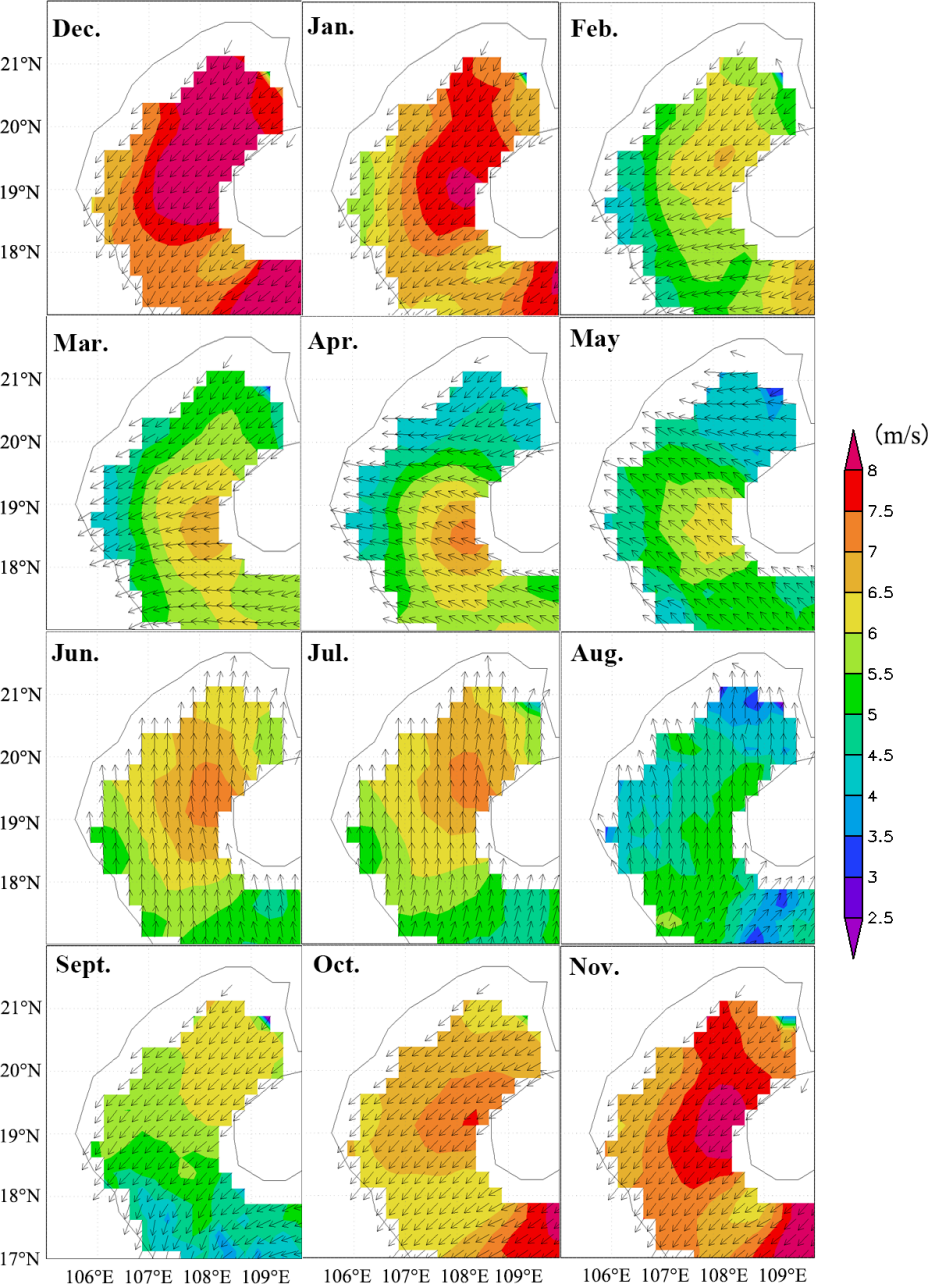
Spatial distribution of monthly averaged climatological wind from 2003 to 2014 in the Beibu Gulf.

The climatological monthly averaged wind velocity over the Beibu Gulf (17°-22°N, 105°40′-110°E) (Figs 3 and 4) shows the typical seasonal features of the East Asian monsoon, with a strong northeast monsoon and weaker southwest monsoon. In the Beibu Gulf, the northeast monsoon (winter season) begins in September with an average of 6.5 m s^-1^; the wind speed has increased until it peaks in December (>8 m s^-1^), and then vanishes the next April. The first appearance of the southwest monsoon is in May and ends in August. The wind speed is relatively higher in the region northwest of Hainan Island than in the other regions at most of the year (Fig 4). Another high-wind area is observed south of Hainan Island during the northeast monsoon; at the same time, there is a low wind southwest of Hainan Island, which might be caused by the blocking effect of Hainan Island when the wind came from the east.

### Spatio-temporal variation of Chl-*a* and SST

**Fig 5:**
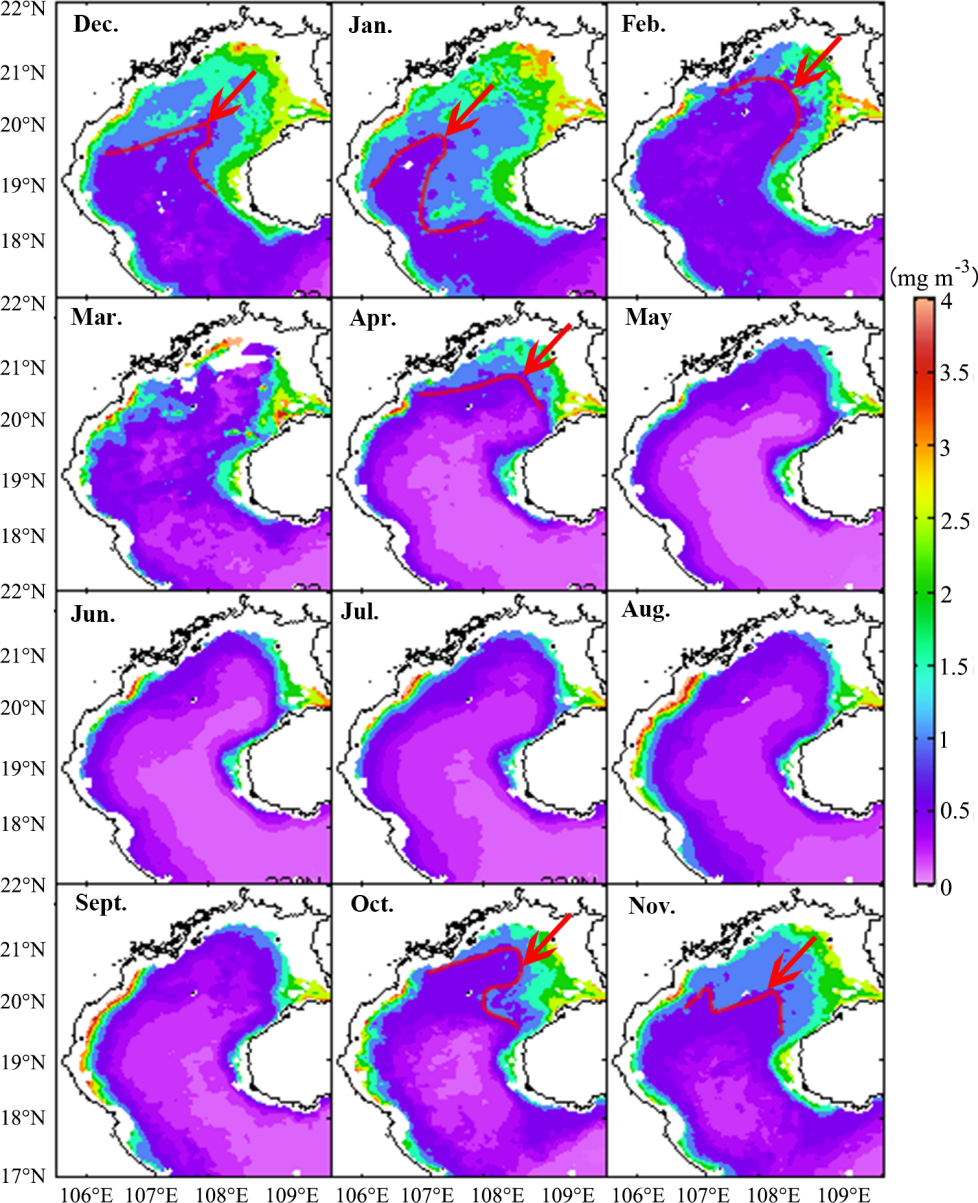
Monthly averaged chl-*a* concentration (mg m^-3^) in the Beibu Gulf. Red arrows and lines show a patch of chl-*a*>1 mg m^-3^ which expands from the northeast region during the northeast monsoon season. Some chl-*a* data were missing for March, and therefore were not available for analysis.

**Fig 6:**
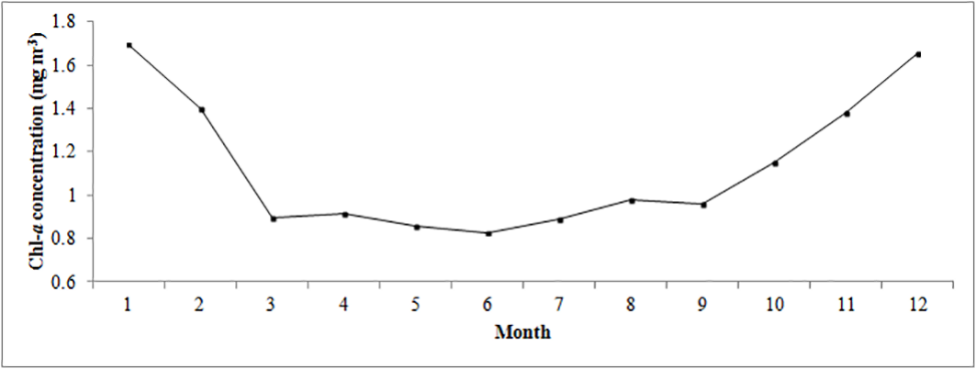
Climatological monthly averaged chl-*a* concentration (mg m^-3^) in the Beibu Gulf during July 2002—December 2014.

The spatio-temporal variation of surface chl-*a* is illustrated by the monthly climatology of MODIS images at a 4-km spatial resolution (Fig 5). The monthly spatial distributions of chl-*a* are generally similar, with relatively higher values along the coastal areas and lower values offshore, consistent with previous studies [7, 17]. In the northwest coastal area, west of the Leizhou Peninsula, west of Hainan Island and in a narrow zone along the coastal water on the Gulf west side, chl-*a* concentrations remained generally high (>2 mg m^-3^) throughout the entire year. In contrast, chl-*a* concentrations were relatively low (<1 mg m^-3^) in deeper (>40 m) water areas and offshore areas outside of the mouth of the Gulf. The distribution of chl-*a* had seasonal patterns with higher values (>1 mg m^-3^) seen during the northeast monsoon and lower values (<0.6 mg m^-3^) seen in spring (Fig 6). During the northeast monsoon, the patch of high chl-*a* concentration along the northeast and west of Hainan Island expanded southwest, increasing gradually starting in October, peaking in January of the next year, and then reducing gradually until April. During the following southwest monsoon, the size of the area with high chl-*a* remained the same. However, the high chl-*a* area along the west coast differed, increasing gradually starting in June at the beginning of the southwest monsoon, peaking in August, and then declining.

**Fig 7:**
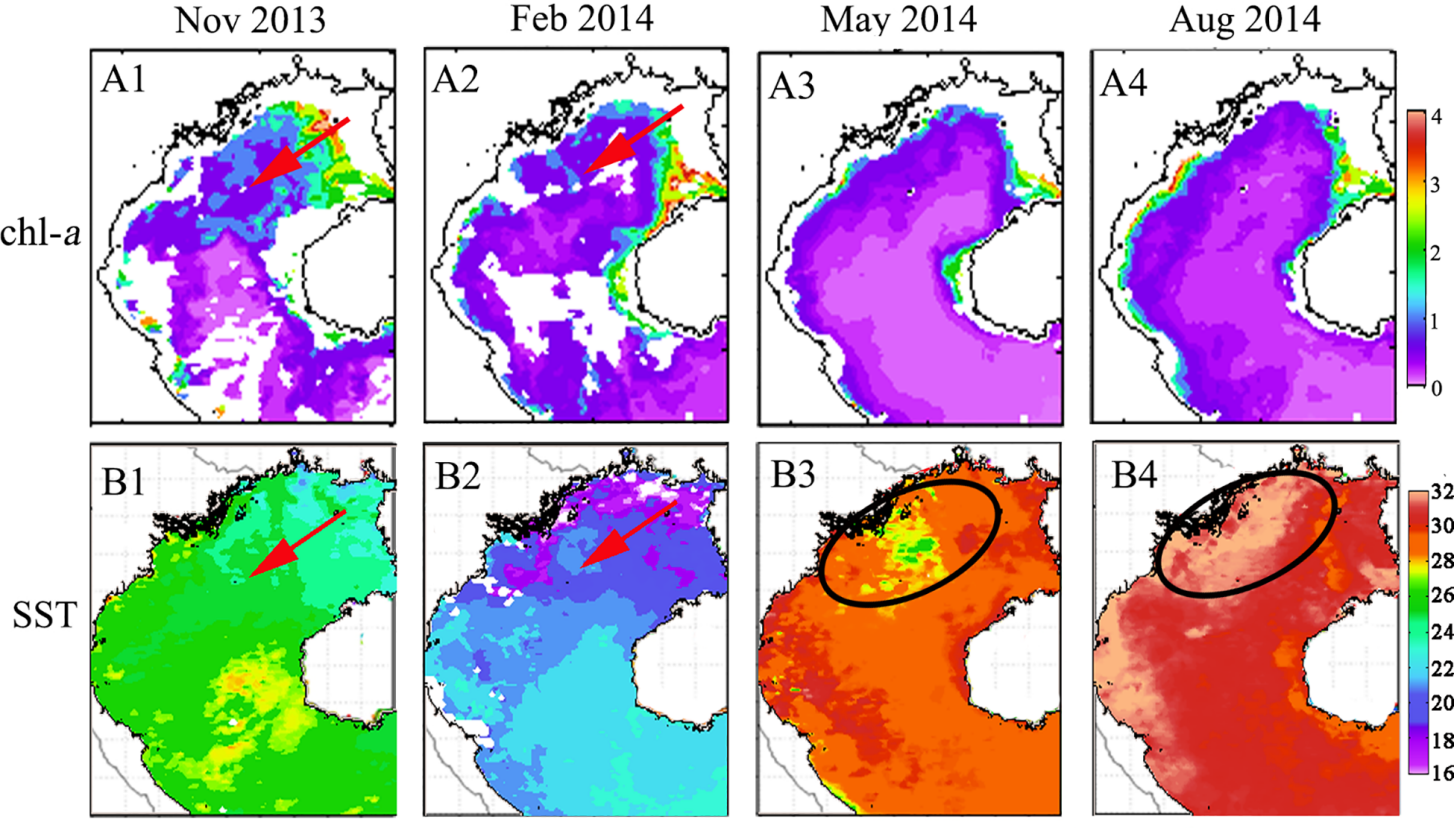
Monthly-averaged chl-*a* (mg m^-3^) and SST (°C) during the field survey cruises in the Beibu Gulf. Red arrows in A1 and A2 show a patch of high chl-*a* concentration increasing towards the southwest. Red arrows in B1 and B2 show that low SST is detected in the same region of high chl-*a* during the northeast monsoon. Red circles in B3 and B4 show that the water temperature in this region was influenced by the Red River discharge in spring and summer. The black arrow in B4 shows a low SST region southwest of Hainan Island in summer.

The comparison of chl-*a* and SST in the Beibu Gulf across the seasons is illustrated by four monthly-mean MODIS images, corresponding to the period of the field survey cruises (Fig 7). In the northeast monsoon season, relatively higher chl-*a* and low SST were observed in the northeast of the Gulf, moving quickly toward the southwest. In autumn (November 2013), SST was low (<23°C) in the northern part and increased gradually southward (>26°C). In the following season (February 2014), SST dropped by about 5°C in the same area, but the spatial distribution was similar to that in autumn, with warm water from the SCS entering the Gulf. From spring (May 2014), SST increased and the distribution was different from that seen during the northeast monsoon. Observed SST was high (>29°C) in most regions of the Gulf, especially in the western regions near 18.5°N, where the SST was generally higher (>30°C). Yet, in a larger area of the northwest coast, lower SST (<28°C) was observed, possibly influenced by the cold waters discharged from the Red River. The heat capacity of the land is smaller than ocean water, so the land temperature changes more quickly, as a result, the river water is hotter in summer and colder in winter compared with ocean water. During summer (August 2014), SST continued to increase, with the largest warming (>32°C) seen in the area along the west coast and west entrance of the Qiongzhou Strait, which might have been caused by the discharge of the Red River and the western current of the Qiongzhou Strait. In the central Gulf, the SST was about 30.5°C, while lower SST (<30°C) was observed around the south and west coasts of Hainan Island.

### Temperature change along three transects

**Fig 8:**
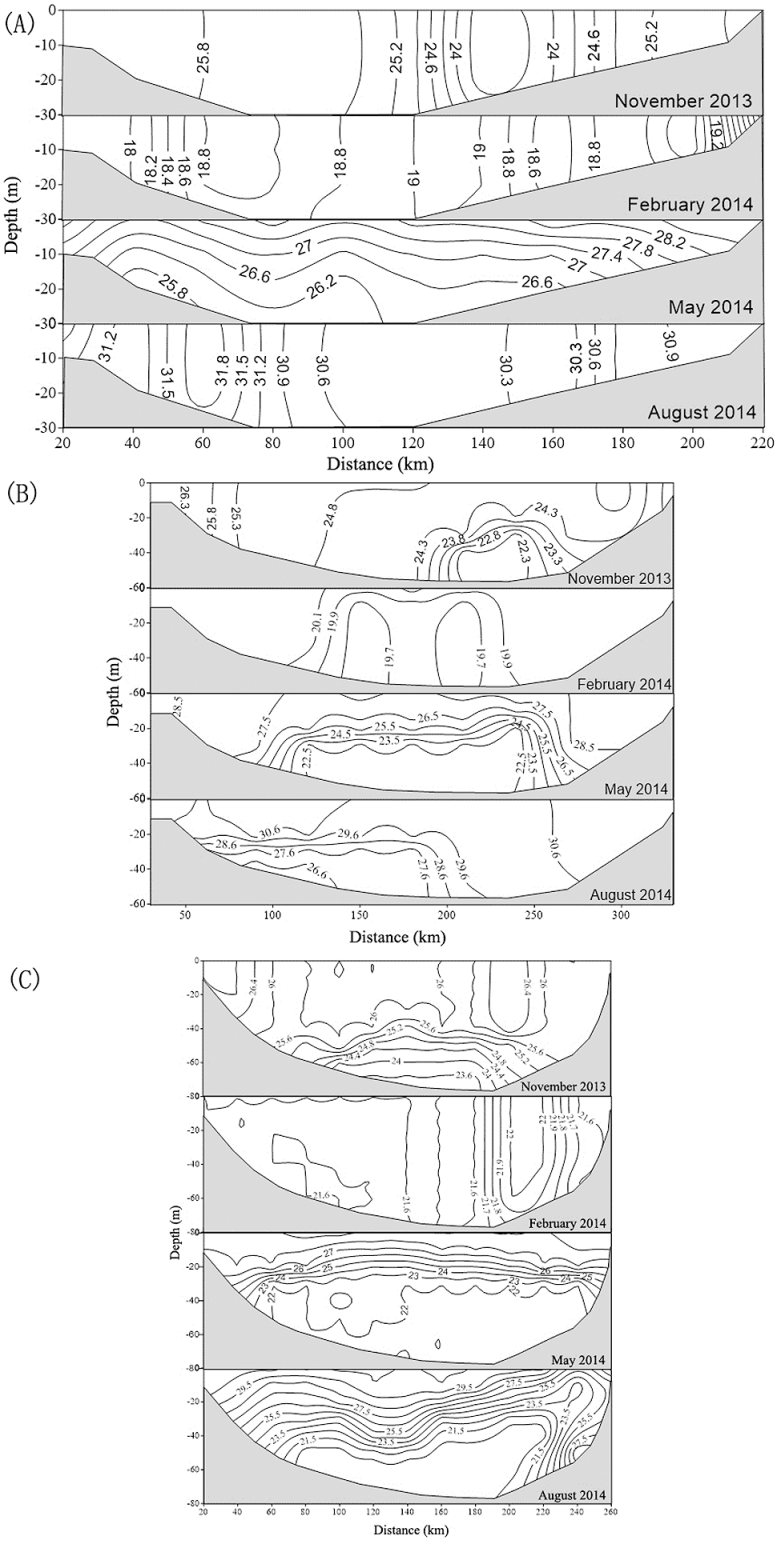
**Profiles of water temperature (°C) along transects T1 (A), T2 (B) and T3 (C).** Abscissa is the distance along the transect (units: km). Ordinate is water depth (units: m).

Water column structure along the three transects (Fig 1: T1, T2 and T3, respectively) in November 2013, February 2014, May 2014, and August 2014 by the CTD and CATSAT data are depicted in Fig 8. During autumn (November 2013), the water was well mixed vertically along T1, T2, and T3 at onshore stations (water depth <30 m) relative to water temperature. In the central Gulf’s deep-water areas (water depth > 40 m along T2 and T3), the sea water was well stratified. In winter, all water columns were well mixed vertically in the entire Gulf. In spring, because of surface warming, the water began to stratify across the Gulf. However, this stratification was not detected in near-shore areas, and shallow water regions were mixed during the following southwest monsoon period. At the same time, cold-water upwelling was observed around the west and south coasts of Hainan Island.

## Discussion

Most recent observations and model results suggest that wind-driven currents dominate in the Beibu Gulf residual currents, then the thermohaline currents and tide-induced residual currents (this relation is more apparent in winter) [22,23]. In winter, a cyclonic circulation prevails in Beibu Gulf, driven by the monsoonal wind [13,24]. The Beibu Gulf summer circulation is cyclonic in the northern gulf and anticyclonic in the southern gulf. The northern circulation is mainly induced by the wind stress curl and the southern circulation is affected by the South China Sea current. Previous studies [13, 24–28] indicated that in the Qiongzhou Strait, the current goes eastward all year around, and it is stronger in winter than in summer. In the southern gulf, both observations and model results displayed a summer upwelling off the southwestern coast of Hainan Island [23]. The above dynamic processes may regulate the seasonal variation of phytoplankton in Beibu Gulf.

### Seasonal dynamics of the mixed-layer depth

**Fig 9:**
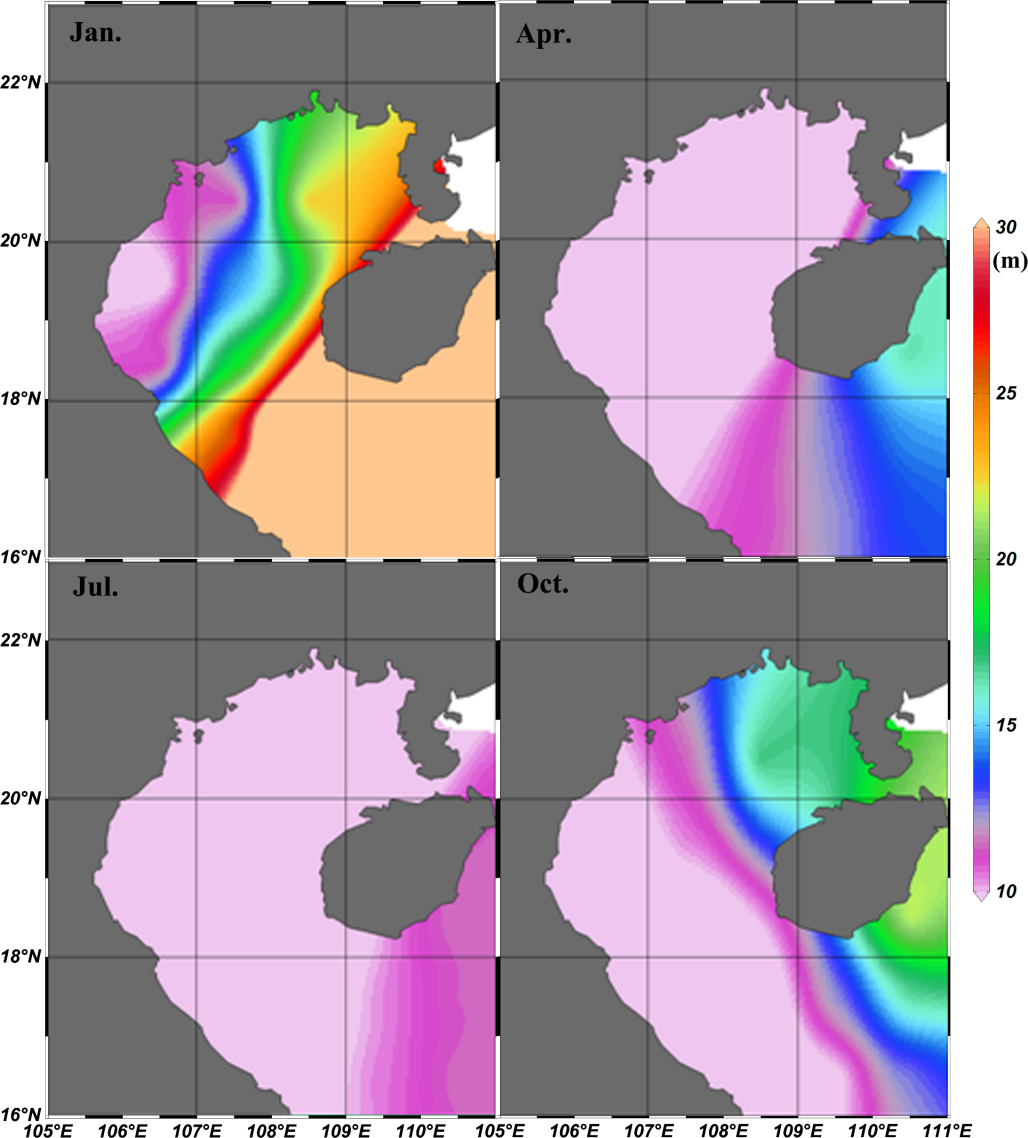
Spatial distribution of monthly-averaged MLD during four seasons from 2002 to 2014 in Beibu Gulf.

**Fig 10:**
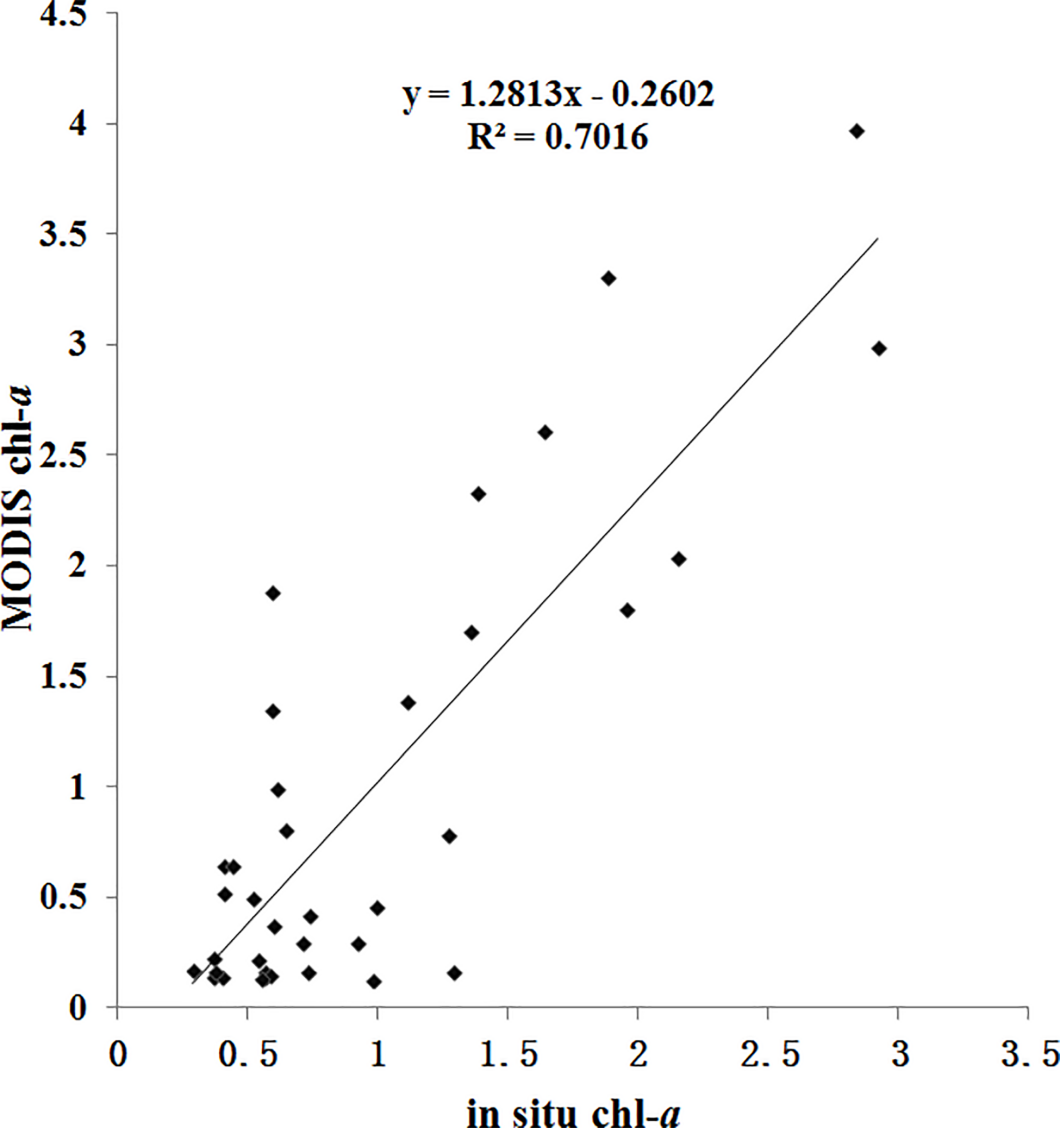
Comparison between MODIS-derived and in situ measurements of chl-*a* in the Beibu Gulf.

Chl-*a* concentration data retrieved from remote sensing imagery in Case-I (open ocean) water produced reasonable results that are comparable to previous studies [29], but the algorithms developed for Case-II (coastal ocean) water can have some inconsistencies, particularly near river mouths and in shallow waters [30,31,32]. Chl-*a* concentrations may be overestimated in Case-II water due to the influence of re-suspended sediments (SM) as well as colored dissolved organic matter (CDOM) [33]. The monthly climatology of MLD indicates the typical seasonal features: strong mixing during the northeast monsoon and weaker mixing in southwest monsoon (Fig 9). In summer, the seawater shows an apparent stratification, and the MLD is less than 13 m in almost all areas of the Beibu Gulf. Next, the vertical mixing enhances in autumn and reaches the peak in winter with MLD deeper than 16 m in most regions, reaching a maximum (MLD>25m) only in the southeastern part of the Gulf, which then begins to decrease in spring. Vertical mixing may bring an abundance of SM to the surface which can influence the remote sensing inversion of chl-*a*. However, comparing the distribution of MLD and the bathymetric chart demonstrates that MLD did not reach the bottom in the areas of Beibu Gulf with a water depth deeper than 20 m, implying that the influence of MLD-derived SM on satellite chl-*a* is limited in most of this area. According to the MLD data, the MLD in the gulf is generally observed shallower than 20 m. Therefore we selected the 20-m depth as one threshold value, to remove influence of sediment from the sea bottom. On the other hand, previous studies have shown that satellite-derived chl-*a* concentration agrees well with in situ measurements including the shallow waters in most of the Beibu Gulf [7,8,34]. In this study, we also evaluate MODIS-derived chl-*a* data in combination with in-situ observations in the Beibu Gulf. The results indicate a high correlation (r = 0.84, p<0.01, RMSE = 0.5751; Fig 10) between the MODIS data and the in situ chl-*a* concentrations, suggesting that MODIS data can be used as a reliable source to effectively identify variability of chl-*a* in the Beibu Gulf.

### Seasonal characteristics of chl-*a* and other oceanic conditions in coastal areas

Phytoplankton production in the ocean is normally limited by nutrient availability and solar radiation [35–37]. In the SCS, light is generally rich in the upper 100 m, and is not the limiting factor for chl-*a* increase due to photosynthesis in the surface layer [38]. It was found that monthly-averaged photosynthetically active radiation (PAR) observed in winter in the SCS was high enough for phytoplankton growth in the upper layers [39]. This suggests that the variation of chl-*a* concentration was probably controlled by availability of nutrients, which was generally regulated by oceanic dynamic processes including currents, mixing, tides, and upwelling. High chl-*a* concentrations in the estuaries and coastal areas (Fig 5) may also be regulated indirectly by an injection of nutrients from coastal currents, including river runoff, and tide-induced currents, as well as effective entrainment mixing of nutrients from the bottom due to shallow depth (Fig 9), to a certain degree. Previous studies showed that the total flux of terrestrial nutrients into the Beibu Gulf is 180,378 tons every year, including 176,997 tons of chemical oxygen demand, 3,277 tons of dissolved organic nitrogen, and 104 tons of dissolved organic phosphorus [40]. Chemical oxygen demand is considered one of the most important quality control parameters of organic pollution in the wastewater [41]. In the coastal area, chemical oxygen demand was confirmed an important role in reflecting the eutrophication status in seawater [42]. As a result of offshore transport effects induced by strong coast currents (>40 cm s^-1^ in velocity) [13], the upwelling of deep water by the coastal current could bring nutrient-laden water closer to the surface and enhance phytoplankton growth. Our results are generally consistent with previous observations in the Beibu Gulf [8, 17], in which the relatively low mean SST of about 22°C in winter directly promoted phytoplankton growth.

### Offshore phytoplankton blooms, and relations to the northeast monsoon

**Fig 11:**
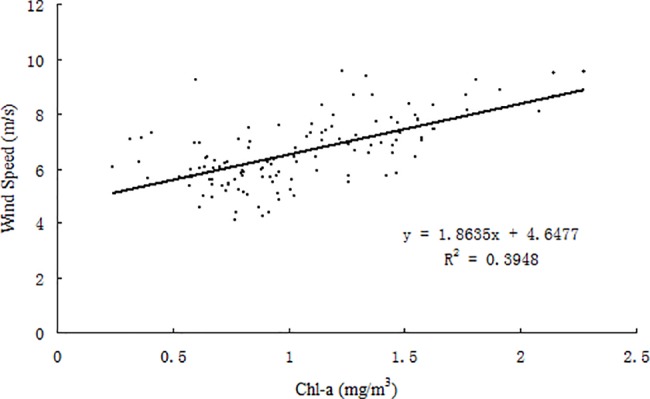
Scatter plot of monthly chl-*a* vs. monthly wind speed from 2002 to 2014 averaged over the Beibu Gulf (105°40′-110°E, 17°-22°N).

The East Asian monsoon regulates annual atmospheric and upper ocean variability in the SCS region [43]. Previous studies have shown that chl-*a* and SST are two key indicators of the response of upper oceanic conditions to atmospheric forcing [44], as chl-*a* concentration increases with decreasing SST in the SCS [1, 45]. In this study, chl-*a* concentration is observed to increase during the northeast monsoon in the Beibu Gulf (Figs 5 and 6), in which high chl-*a* concentrations (>2 mg m^-3^) along the coast in the northeastern Gulf extend towards the southwest after the onset of the northeast monsoon. The gradually increasing size of a high chl-*a* region coincides with increasing wind speed (Figs 4–7). Correlation analysis indicates that the change of monthly chl-*a* level in the Gulf was closely related to monthly wind speed (r = 0.63, p<0.01; Fig 11), i.e., higher chl-*a* is observed under stronger wind conditions. Spatial variability of higher chl-*a* was observed, coinciding with lower SST in the northeast Gulf (Fig 7). Comparison of monthly chl-*a* and SST during the northeast monsoon shows that higher chl-*a* concentrations correspond to lower SST in the same area, and vice versa. Low SST often reflects strong vertical mixing in offshore regions [46]. In the SCS, nutrients generally increase with depth below the surface [47]. During the northeast monsoon, due to surface cooling (Fig 7) and strong vertical mixing/entrainment (Fig 9), cold water with abundant nutrients was easily brought up from the bottom and, therefore, chl-*a* concentrations became higher, especially in winter. Further validation of the winter monsoon’s contribution to increased mixing can be seen by examination of the temperature profiles in Fig 9 during different seasons, which shows a homogenous vertical structure of temperature during November 2013 and February 2014 compared to strongly stratified conditions during the summer season (May and August 2014). This vertical homogeneity is most striking in the shallowest waters analyzed (Transect T1, Fig 8A).

**Fig 12:**
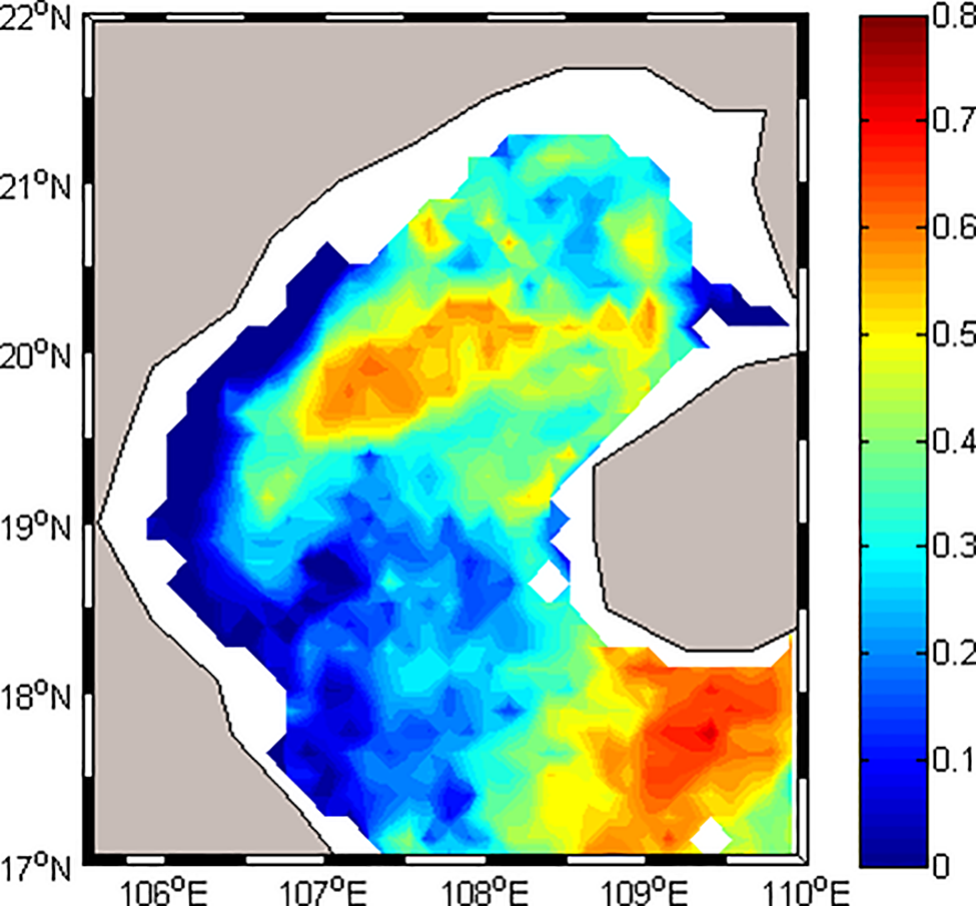
The correlation map of Windsat/ wind speed and MODIS/ chl-*a* data over the Beibu Gulf, based on 12-year time series (Feb 2003 to Dec 2014), at a resolution of 0.25° by 0.25°.

The correlation between wind speed and chl-*a* in the whole Gulf is analyzed to evaluate the contribution of the monsoon to the distribution of chl-*a* in the Beibu Gulf (Fig 12). Correlations are relatively high (r>0.7, P<0.005) in the middle of the northern Gulf and southern Hainan Island, and it decreases around the two regions and reaches the minimum near the west coast. In the region of 17°-19°N, 106°-108°E, the correlations are relatively low (r<0.3). Previous studies reported the strongest currents (>40 cm s^-1^) in the Qiongzhou Strait and the area southwest of Hainan Island [48]. As a result, in coastal areas, the contribution of wind to chl-*a* increase is relatively low, and the change of chl-*a* is due to the influence of tidal currents, runoff from rivers, and shallow depths. Conversely, in offshore areas, where the other prominent nutrient sources (e.g., terrestrial) are absent, so the wind is the main factor controlling mixing and influencing the distribution of chl-*a* (r>0.6, p<0.05). In the region of 17°-19°N, 106°-108°E, the correlations are relatively low (r<0.3). This can be ascribed to two factors. First is the runoff by the Red River, Majiang River and Dajiang River [13], which becomes the main reason supplying significant amounts of nutrients for this region. The other reason is that the wind speed remains low during all seasons and induces very little vertical mixed in this region (Figs 4 and 9).

### Seasonal SST behavior near Hainan Isalnd

In summer, SST increased in the Beibu Gulf, particularly in coastal areas (Figs 7 and 8). However, lower SST was observed in the southwest coastal region of Hainan Island. Fig 8C depicts that there was a shoaling of isotherms at the east end of the profile, suggesting the presence of cold water upwelling in the region east of Hainan Island. Upwelling west of Hainan Island was also found in previous studies [49–51]; in which the tidal mixing front was confirmed as the main factor inducing upwelling, especially in summer. The southwest monsoon induces downwelling, which competes with the front-induced upwelling. Concurrently, the diluted water of the Changhua River may be a contributing factor enhancing the upwelling in summer. The Changhua River is the second largest river flowing westward into the Beibu Gulf from Hainan Island, with an annual flow of 50–55×10^8^ m^3^, and a peak flow in summer [52]. Due to the strong runoff from the Changhua River and the topography of the coastal area, discharge from the river was easily transported offshore, which was the auxiliary driving force to bring up deep cold water. Furthermore, injection of nutrients from river runoff and upwelling likely triggered higher chl-*a* levels around western Hainan Island.

## Conclusion

The spatial patterns of monthly chl-*a* concentrations are generally similar, with relatively high values along the coastal areas and lower values offshore. The time series of chl-*a* presents also evidently seasonal variation in most years, with higher values (>1 mg m^-3^) during the northeast monsoon and lower values (<0.6 mg m^-3^) in spring. Chl-*a* concentration in the offshore Beibu Gulf is controlled by nutrients, which is largely influenced by wind-induced mixing and upwelling (wind vs. chl-*a*: r = 0.63, p<0.01), as well as currents and so on. High chl-*a* in the estuaries and coastal areas may be probably due to the nutrients injection from upwelling and coastal currents as well as river runoff, and mixing. Higher chl-*a* in the southwest coastal region of Hainan Island in summer may be due to the upwelling driven by the southwesterly monsoonal winds and the runoff from the Changhua River, as suggested by lower SST in the region.

## Supporting information

S1 File Data of Fig 2 Comparison between CATSAT-derived and in-situ measurements of water temperature. (XLSX)

S2 File Data of Fig 6. Climatological monthly averaged chl-a concentration (mg m^-3^) in the Beibu Gulf during July 2002—December 2014.
(XLS)

S3 File Data of Fig 8. Profiles of water temperature (°C) along transects T1 (A), T2 (B) and T3 (C).
(XLS)

S4 File Data of Fig 10. Comparison between MODIS-derived and in situ measurements of chl-a in the Beibu Gulf.
(XLS)

S5 File Data of Fig 11. Scatter plot of monthly chl-a vs. monthly wind speed from 2002 to 2014 averaged over the Beibu Gulf.
(XLS)

S6 File Data of Fig 12. The correlation map of Windsat wind speed and MODIS chl-a data over the Beibu Gulf, based on 12-year time series (Feb 2003 to Dec 2014), at a resolution of 0.25° by 0.25°.
(XLSX)

S7 File The geographical locations and dates of in situ sampling. In situ chl-*a* and water temperature profiles were collected at the nine stations in four cruise surveys conducted in November 2013, February 2014, May 2014, and August 2014 in the study area shown in Fig 1.
(DOCX)

## References

[1] Liu KK, Chao SY, Shaw PT, Gong GC, Chen CC, Tang TY. Monsoon-forced chlorophyll distribution and primary production in the South China Sea: observations and a numerical study. Deep-Sea Research. 2002; 149:1387–1412.

[2] Shaw P T, Chao SY. Surface circulation in the South China Sea. Deep Sea Research. 1994; 41(11/12):1663–1683.

[3] Jing ZY, Qi YQ, Hua ZL, Zhang H. Numerical study on summer upwelling system in the northern continental shelf of the South China Sea. Continental Shelf Research. 2009; 29: 467–478.

[4] Tang DL, Kawamura H, Dien TV, Lee MA. Offshore phytoplankton biomass increase and its oceanographic causes in the South China Sea. Marine Ecology Progress Series. 2004; 268: 31–41.

[5] Liu G, Chai F. Seasonal and interannual variability of primary and export production in the South China Sea: A three‐dimensional physical‐biogeochemical model study. Ices Journal of Marine Science. 2009; 66 (2): 420–431.

[6] Chai F, Liu GM, Xue H J, Shi L, Chao Y, Tseng CM, et al Seasonal and interannual variability of carbon cycle in South China Sea: A three‐dimensional physical‐biogeochemical modeling study. Journal of Oceanography. 2009: 65(5):703–720.

[7] Ning X, Chai F, Xue H. Cai Y. Liu C, Shi J, Physical-biological oceanographic coupling influencing phytoplankton and primary production in the South China Sea. Journal of Geophysical Research-Oceans. 2004; 109 (C10): 215–255.

[8] Tang D L, Kawamura H, Lee M A, Dien TV. Seasonal and spatial distribution of chlorophyll-a concentrations and water conditions in the Gulf of Tonkin, South China Sea. Remote Sensing of Environment. 2003; 85(3):475–483.

[9] Botsford LW, Lawrence CA, Dever EP, Hastings A, Largier J. Effects of variable winds on biological productivity on continental shelves in coastal upwelling systems. Deep-Sea Research Part II -Topical Studies in Oceanography. 2006; 53 (25–26): 3116–3140.

[10] Chao SY, Shaw PT, Wu SY. Deep water ventilation in the south China Sea. Deep-Sea Research Part I. 1996; 43:445–466.

[11] Chen CC, Shiah FK, Chung SW, Liu KK. Winter phytoplankton blooms in the shallow mixed layer of the South China Sea enhanced by upwelling. Journal of Marine Systems. 2006; 59 (1–2), 97–110.

[12] Liu ZL, Ning XR, Cai YM. Distribution characteristies of size-fractionated chlorophyll-*a* and productivity of phytoplankton in the Beibu Gulf. Acta Oceanologica Sinica. 1998; 20 (1): 50–57.

[13] Zu TT. Analysis of the current and its mechanism in the Gulf of Beibu. Ocean University of China. 2005.

[14] Van Maren DS, Hoekstra P. Dispersal of suspended sediments in the turbid and highly stratified Red River plume. Continental Shelf Research. 2005; 25(4): 503–519.

[15] Van Maren DS. Water and sediment dynamics in the Red River mouth and adjacent coastal zone. Journal of Asian Earth Sciences. 2007; 29(4): 508–522.

[16] Wu YC, Guo F, Huang LF, Huang FP. Distribution Characteristics of Chlorophyll a Content in the Beibu Gulf. Guangzhou Chemical Industry. 2014; 42(8): 144–146.

[17] Huang YC, Li Y, Shan H, Li YH. Seasonal Variations of Sea Surface Temperature, Chlorophyll a and Turbidity in Beibu Gulf, MODIS Imagery Study. Journal of Xiamen University (Natural Science). 2008; 47(6): 856–863.

[18] Parsons TR. A manual of chemical & biological methods for seawater analysis. Elsevier, 2013.

[19] Shen CY, Shi P, Zhao H, Zhang YY, Dong JD. Spatio-temporal distribution of the chlorophyll a concentration derived from MODIS satellite remote sensing and in situ observations in the Sanya Bay of China. Internatrional Journal of Remote Sensing, 2014; 35(11–12): 4127–4137.

[20] Levitus S. Climatological Atlas of the World Ocean, NOAA Prof. Pap. 1982; 13, 173 pp., U.S. Govt. Printing Off, Washington, D.C.

[21] Monterey G, Levitus S. Seasonal Variability of Mixed Layer Depth for the World Ocean. NOAA Atlas NESDIS 14, U.S. Gov. Printing Office, Wash., D.C, 96 pp. 87 figs. 1997.

[22] Sun HL, Huang WM, Zhao JS. Three-dimensional numerical simulation of tide-induced, wind-driven and thermohaline residual currents in the Beibu Bay. Oceanol. Limnol. 2001; 32: 561–568.

[23] Gao JS, Wu GD, Ya HZ. Review of the circulation in the Beibu Gulf, South China Sea. Continental Shelf Research, 2017; 138: 106–119.

[24] Gao JS, Chen B. Analysis on characteristics and formation mechanism of the winter boreal circulation in the Beibu Gulf. Guangxi Science. 2014; 21: 64–72.

[25] Yuan SY, Deng JZ. Numerical research on the circulation in the Beibu Gulf. Nanhai Yanjiu Yu Kaifa, 1999; 2: 41–6.

[26] Manh DV, Yanagi T. A study on the residual flow in the Beibu Gulf. Journal of Oceanography. 2000; 56: 59–68.

[27] Xia HY, Li SH, Shi MC. Three-D numerical simulation of wind-driven current and density current in the Beibu Gulf. Acta Oceanol. Sin. 2001; 20: 455–472.

[28] SuJL Yuan YL. Hydrography of China Seas. China Ocean Press, Beijing, 2005.

[29] Gordon HR. Radiometric considerations for ocean color remote sensors. Applied Optics.1990; 29: 3228–3236. doi: 10.1364/AO.29.003228 20567403 10.1364/AO.29.003228

[30] Mélin F, Zibordi G, Berthon J F. Assessment of satellite ocean color products at a coastal site. Remote Sensing of Environment, 2007; 110(2):192–215.

[31] Kahru M, Kudela R, Anderson C, Manzanosarabia M, Mitchell B. Evaluation of Satellite Retrievals of Ocean Chlorophyll-a in the California Current. Remote Sensing, 2014, 6(9):8524–8540.

[32] Shang SL, Lee ZP, Shi LH, Lin G, Wei GM, Li XD. Changes in water clarity of the Bohai Sea: Observations from MODIS. Remote Sensing of Environment, 2016; 186:22–31.

[33] IOCCG. Remote Sensing of Ocean Color in Coastal, and Other Optically-Complex Waters; Sathyendranath S, editors. Reports of the International Ocean-Color Coordinating Group, No. 3; IOCCG: Dartmouth, NS, Canada, 2000.

[34] Chen B. A preliminary study on the formation and characteristics of the water system in the Beibu Gulf. Journal of Guangxi Academy of Sciences. 1986; 2 (2): 92–95.

[35] Tang DL, Kawamura H, Doan-Nhu H, Takahashi W. Remote sensing oceanography of a harmful algal bloom off the coast of southeastern Vietnam. Journal of Geophysical Research Oceans. 2004; 109 (3): 325–347.

[36] Siegel H, Ohde T, Gerth M, Lavik G, Leipe T. Identification of coccolithophore blooms in the SE Atlantic Ocean off Namibia by satellites and in-situ methods. Cont. Shelf Res. 2007; 27 (2): 258–274.

[37] Dwivedi RM, Raman M, Babu KN, Singh SK, Vyas NK, Matondkar SGP. Formation of algal bloom in the northern Arabian Sea deep waters during January-March: a study using pooled in situ and satellite data. Int. J. Remote Sens. 2008; 29 (15): 4537–4551.

[38] Zhao H, Tang DL. The Spatial Distribution of Chlorophyll-a and Its Responses to Oceanographic Environments in The South China Sea. Advances in Geoscience.2006; 5(2):7–14.

[39] Chen CC, Shiah FK, Chung SW, Liu KK. Winter phytoplankton blooms in the shallow mixed layer of the South China Sea enhanced by upwelling. Journal of Marine Systems. 2006; 59: 97–110.25. Wei MX, He BM. An analysis of the environmental characteristics and nutrient status of beihai bay. Transactions of Oceanology and Limnology. 2003; (4): 95–100.

[40] Wei MX, He BM. A Preliminary analysis of nitrate content in the Beibu-Gulf. Marine Sciences. 1988; 4:46–52.

[41] Tasnim A. Estimation of Chemical Oxygen Demand in WasteWater using UN-VIS Spectroscopy. Simon Fraser University. 2015.

[42] Sun H, Zhang QF, Tu JB, Ma YY, Yin CL, Wang B. Distribution and influencing factors of COD, along with the relationship with eutrophication in Tianjin coastal area. Marine Environmental Science. 2017; 36(3): 343–371

[43] Wang B, Huang F, Wu Z, Yang J, Fu X, Kikuchia K. Multi-scale climate variability of the South China Sea monsoon: a review. Dynamics of Atmospheres and Oceans. 2009; 47(1–3):15–37.

[44] Tan SC, Wang H. The transport and deposition of dust and its impact on phytoplankton growth in the Yellow Sea. Atmospheric Environment. 2014; 99: 491–499.

[45] Yang YJ, Xian T, Sun Fu YF. Summer Monsoon Impacts on Chlorophyll-a Concentration in the Middle of the South China Sea: Climatological Mean and Annual Variability. Atmospheric and Oceanic Science Letters. 2012; 5(1):15–19.

[46] Martin MC, Villanoy CL. Sea Surface Variability of Upwelling Area Northwest of Luzon, Philippines, Dynamic Planet. Springer Berlin Heidelberg 2007; 130: 84–87.

[47] Ye HJ, Sui Y, Tang DL, Afanasyev YD. A subsurface chlorophyll a bloom induced by typhoon in the South China Sea. Journal of Marine Systems. 2013; 128:138–145.

[48] Huang QZ, Wang WZ, Chen J C. Tides tidal currents and storm surge set-up of South China Sea. In Zhou D, Liang YB, Zeng CK, editors. Oceanology of China Seas; 1994. pp. 113–122.

[49] Chen ZZ, Hu JY, Sun ZY, Zhu J. Sectional features of temperature and salinity in Beibu Gulf during July-August, 2006 Hu JY, Yang SY. Marine scientific research papers in Beibu Gulf. Beijing: The Ocean Publishing Company, 2008.

[50] Lu XG, Qiao FL, Wang GS, Xia CS, Yuan YL. Upwelling off the west coast of Hainan Island in summer: Its detection and mechanisms. Geophysical Research Letters, 2008, 35(2):196–199.

[51] Chen ZH. Numerical simulation on seasonal variation of ocean circulation and its dynamic mechanism in the Beibu Gulf. Doctor dissertation. Ocean University of China. 2013

[52] Xiao M. The Application of GIS in Watershed ecological quality evaluation-a case study in Changhuajiang downstream. Hainan University 2011.

