# Supplementary material for: Influence of monsoonal winds on chlorophyll-α distribution in the Beibu Gulf
Source: PLoS One. 2018 Jan 12;13(1):e0191051. doi: 10.1371/journal.pone.0191051 (PMC5766212; doi:10.1371/journal.pone.0191051)
Supplement: S7 File — In situ chl-a and water temperature profiles were collected at the nine stations in four cruise surveys conducted in November 2013, February 2014, May 2014, and August 2014 in the study area shown in Fig 1. (DOCX) [file pone.0191051.s007.docx]

**
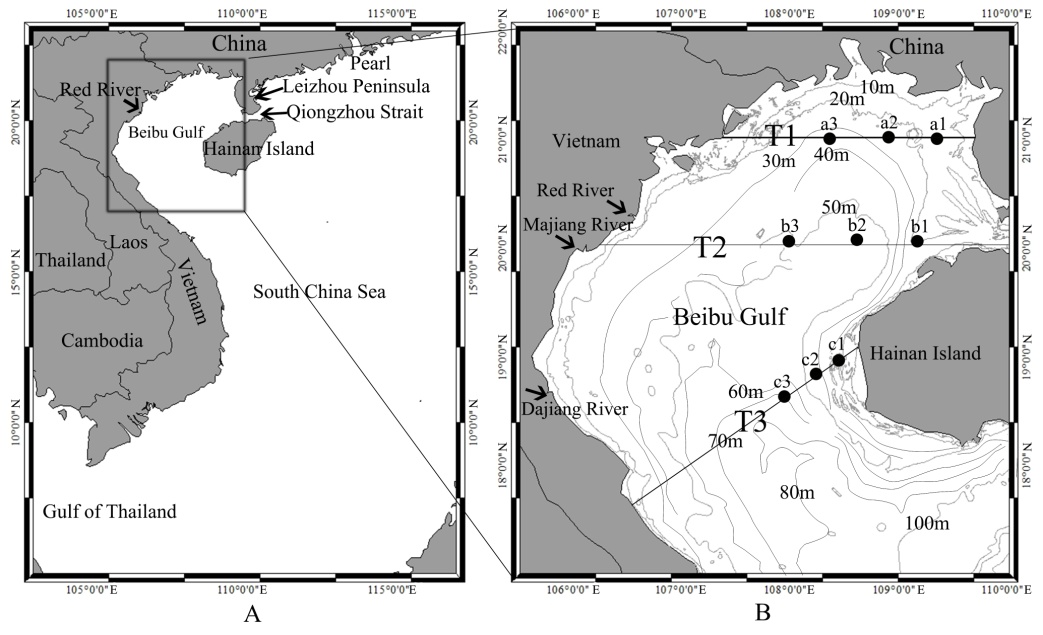
**

**Figure 1. Sampling stations**

**Sampling stations:**

a1 (109.321839°E, 21.048581°N)

a2 (108.820929°E, 21.040791°N)

a3 (108.219711°E, 21.042211°N)

b1 (109.145139°E, 20.032882°N)

b2 (108.549878°E, 20.019479°N)

b3 (107.92925°E, 20.005580°N)

c1 (108.451163°E, 18.984222°N)

c2 (108.260206°E, 18.850869°N)

c3 (107.968687°E, 18.654920°N)

**Sampling dates:**

24-31, November 2013

2-8, February 2014

13-21, May 2014

17-25, August 2014
